# Reduced heart rate response to exercise in patients with type 2 diabetes

**DOI:** 10.3389/fcvm.2025.1446675

**Published:** 2025-03-31

**Authors:** Jingfeng Lou, Hongmei Lang, Yuhan Xia, Hui Jiang, Kun Li, Xingping Zhang

**Affiliations:** ^1^ Department of General Medicine, Chengdu Second People’s Hospital, Chengdu, China; ^2^ Department of Endocrinology, North Sichuan Medical College, Nan Chong, China; ^3^ Department of General Medicine, Chengdu Second People's Hospital, Clinical Medical College of Chengdu Medical College, Chengdu, China; ^4^ Department of General Medicine, Qiyang People’s Hospital, Yongzhou, China; ^5^ Department of General Medicine, Hongpailou Community Health Care Center, Chengdu, China

**Keywords:** heart rate response, chronotropic index, cardiopulmonary exercise testing, type 2 diabetes, correlation

## Abstract

**Background:**

Recent studies have found that heart rate response is impaired in patients with type 2 diabetes. However, it remains unclear how chronotropic competence changes in these patients and which chronotropic index is more closely related to type 2 diabetes. This study aims to investigate the changes in chronotropic competence in type 2 diabetes and compares the association of two different chronotropic indices with type 2 diabetes.

**Patients and methods:**

Patients who underwent cardiopulmonary exercise testing at the Chengdu Second People's Hospital from October 2022 to October 2023, we included. Logistic regression was used to analyze the relationship between chronotropic indices and type 2 diabetes, comparing which of the two chronotropic indices is more closely related to type 2 diabetes.

**Results:**

A total of 166 patients were included in our study, of which 42.8% had type 2 diabetes and 57.2% did not have type 2 diabetes. After adjusting for confounders, the OR for chronotropic index 1 with type 2 diabetes was 0.001 (95% CI: 0.0001–0.521, *P* = 0.03), and the OR for chronotropic index 2 with type 2 diabetes was 0.665 (95% CI: 0.479–0.923, *P* = 0.015), both showing a negative correlation with type 2 diabetes. When chronotropic index 2 was included in the model as quartiles, it still showed a negative correlation with type 2 diabetes (OR: 0.388; 95% CI: 0.173–0.869; *P* = 0.021), while chronotropic index 1 showed no significant correlation.

**Conclusion:**

Heart rate response is reduced in patients with type 2 diabetes, and a low chronotropic index 2 is independently associated with type 2 diabetes.

## 1 Introduction

Type 2 diabetes (T2DM) is a significant cardiovascular risk factor and increases the risk of mortality. Globally, diabetes affects 6.1% of the population, with T2DM constituting 96% of these cases, thereby representing a significant global health challenge (1). Patients with T2DM may develop several complications, including autonomic neuropathy, which is a dysfunction of the sympathetic and parasympathetic nervous systems (2, 3). The entire process of physical activity reflects the dynamic balance between the parasympathetic and sympathetic nerves. At rest, the maintenance of resting heart rate primarily relies on the parasympathetic nervous system. During the recovery period after exercise, the parasympathetic nervous system is gradually activated, and the sympathetic nervous system is suppressed, leading to a gradual decrease in heart rate (2). However, during exercise, the sympathetic nervous system is activated, the parasympathetic nervous system is inhibited, and the heart rate increases. The increase in heart rate reflects the balance of parasympathetic and sympathetic nervous system function, known as chronotropic competence, and how this competence changes in patients with T2DM is not yet well understood.

Chronotropic incompetence, characterized by the heart's failure to adequately increase its rate in response to activity or demand, is common among patients with cardiovascular diseases. It reduces exercise tolerance, adversely affecting quality of life, and serves as an independent predictor of major cardiovascular events and overall mortality (4). The mechanisms underlying impaired chronotropic function in T2DM remain unclear; however, studies suggest it may be associated with hyperglycemia, dyslipidemia, and abnormalities in insulin signaling pathways (5, 6). Cardiopulmonary exercise testing (CPET), as a vital diagnostic and assessment tool, can evaluate and measure the heart's chronotropic competence. In CPET, chronotropic incompetence is defined as the failure to reach 85% of the age-predicted maximal heart rate, or a low chronotropic index (heart rate adjusted to the MET level) (7, 8). However, there is no definitive, unified standard for calculating chronotropic competence. Some studies represent it as (HRpeak-HRrest) (220-age-HRrest) (9), while the 2012 CPET guidelines suggest using the change in heart rate per increase of 1 MET to assess chronotropic competence (10). It remains unclear which indicator is more closely related to chronotropic competence in patients with T2DM.

Although the relationship between T2DM and cardiovascular complications has been extensively explored, the variations in chronotropic competence among patients with T2DM remain unclear. It is crucial to determine which of the previously mentioned calculation methods is more accurate for assessing chronotropic competence in these patients. Therefore, this study aims to investigate the relationship between T2DM and chronotropic competence, and to compare the associations of two different chronotropic indices with T2DM.

## 2 Materials and methods

### 2.1 Participants

In this study, we included adult patients who underwent CPET at the Chengdu Second People's Hospital from October 2022 to October 2023. We excluded the following patients: (1) those who did not follow the protocol; (2) those who were unable to complete submaximal exercise; (3) patients taking β-blockers and (4) patients under the age of 18. Ultimately, 166 patients (mean age 56 years old) were included in our study, comprising 71 individuals with diabetes and 95 without diabetes. Among them, middle-aged and older patients constituted the majority, comprising 83% of the total individuals (the specific age distribution of participants provided in Supplementary Figure S1). This study was approved by the Ethics Committee of the Chengdu Second People's Hospital, and all participants provided written informed consent.

### 2.2 Clinical data

All data were obtained from the database of the Chengdu Second people's Hospital. Demographic and clinical information included: gender, age, height, weight, waist circumference, smoking history, hypertension, coronary artery disease, and heart failure. BMI was calculated as weight divided by the square of height. Smoking history was defined as having smoked continuously or cumulatively for more than six months. T2DM was diagnosed based on at least one of the following criteria: use of diabetes medications or insulin, a physician diagnosis of T2DM, fasting blood glucose ≥7 mmol/L, or a 2-hour oral glucose tolerance test blood glucose ≥11.1 mmol/L. Hypertension is defined as having a blood pressure greater than 140/90 mmHg on at least three separate occasions, or a history of hypertension. Coronary artery disease was defined as a history of stable or unstable angina, acute myocardial infarction, or ischemic cardiomyopathy. Heart failure was defined as being classified as NYHA II or higher, or having a history of decompensated heart failure. The test indicators include: blood glucose, troponin, NT-proBNP, total cholesterol, and low-density lipoprotein.

### 2.3 Exercise test protocol

The CPETs were conducted at the Chengdu Second People's Hospital, with all testing environments meeting the required standards, including pre-test gas calibration. According to the guidelines of the American College of Cardiology/American Heart Association, all participants underwent symptom-limited cardiopulmonary bicycle exercise testing using the standard Ramp protocol (11, 12). The Ramp protocol is a linear incremental exercise test where the workload is gradually increased, with the power incrementing progressively every second. The test includes a 3-minute rest period, a 3-min warm-up period, followed by exercise, with the duration of the exercise varying according to the patient's condition. The recovery period lasts 6–8 min. During the exercise, the pedaling rate is maintained at 60–70 revolutions per minute. The workload is increased progressively every minute based on a predefined power increment per minute. The power increment per second is calculated by dividing the predefined power increment per minute by 60 s. Endpoints for exercise testing included: a rating of perceived exertion (6–20 scale) >17 (very hard) or a peak respiratory exchange ratio (RER) >1.15; participant request to stop the test due to volitional fatigue; systolic blood pressure ≥240 mmHg or diastolic blood pressure ≥110 mmHg; significant chest discomfort during exercise; severe arrhythmias; or horizontal or downsloping ST-segment depression greater than 2 mm or ST-segment elevation greater than 1 mm.

### 2.4 Exercise test variables

Resting period blood pressure and heart rate were measured after at least 5 min of rest. Peak systolic blood pressure, peak diastolic blood pressure, peak heart rate, and METs were recorded at peak VO2. Heart rate recovery was defined as the peak heart rate minus the heart rate after 1 min of recovery. CI 1 was calculated as (peak HR - resting HR)/(220 - age - resting HR). CI 2 was calculated as (peak HR - resting HR)/(peak METs - resting METs). Peak VO2 was defined as the highest 10-second average VO2 during the final stage of a symptom-limited exercise test. The VE/VCO2 slope represented gas exchange from rest to the peak of exercise. Heart rhythm was monitored using continuous 12-lead electrocardiography.

### 2.5 Statistical analysis

Continuous variables with normal distributions were described as mean ± standard deviation, while those with non-normal distributions were expressed as median and interquartile range. Categorical variables were presented as frequencies and percentages. To compare continuous variables between different groups, the two-sided independent or paired *t*-test was used for normally distributed data; for non-normally distributed data, the Wilcoxon rank-sum test was employed. Pearson's Chi-square test was used to compare frequency distributions. To explore the relationship between diabetes and various indices of chronotropic competence, univariate logistic regression analysis was conducted in the first model. Subsequently, we performed multivariate logistic regression analysis, including all variables that were significantly associated with T2DM in the univariate analysis. In the second model, we adjusted for age, gender, BMI, and smoking history; in the third model, we additionally adjusted for hypertension, heart failure, and coronary artery disease, beyond the factors in the first model; in the fourth model, besides the factors adjusted in the first two models, we also adjusted for blood glucose, NT-proBNP, VO2/Kg, and VE/VCO2 slope. All statistical analyses were performed using SPSS software, version 26, and two-sided probability values <0.05 were considered statistically significant.

## 3 Results

### 3.1 Participant characteristics

**Figure 1:**
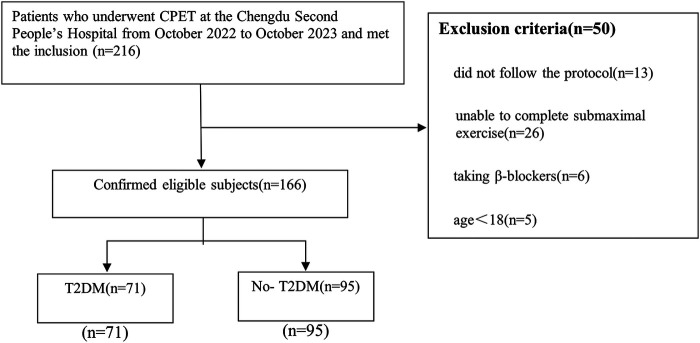
Flow chart of inclusion and exclusion.

Initially, we collected data from 216 patients, of which 166 met the criteria and were included in our study (Figure 1). Among these patients, 71 (42.8%) had T2DM and 95 (57.2%) did not. Baseline data and test results are shown in Table 1. The average age was 55.9 ± 14.4 years, approximately 51.8% were female, the BMI was 24.4 ± 4.0 kg/m^2^, waist circumference was 85.8 ± 11.1 cm, and 21.7% had a history of smoking. The T2DM group had higher blood glucose and lower NT-proBNP, while there were no significant differences between the two groups in terms of troponin, total cholesterol, and low-density lipoprotein. The detailed characteristics of the study population are shown in Table 1.

**Table 1:** Baseline characteristics of the patients with and without T2DM.

Variable	Overall (166)	2T2DM (71)	No-T2DM (95)	*P*-value
Age (years)	55.9 ± 14.4	57.9 ± 13.2	54.4 ± 15.1	0.11
Sex (% female)	86 (51.8)	26 (36.6)	60 (63.2)	0.001
BMI (kg × m^−2^)	24.4 ± 4.0	24.2 ± 4.0	24.6 ± 4.8	0.66
Waist circumference (cm)	85.8 ± 11.1	86.3 ± 11.9	85.4 ± 10.5	0.67
Smoking history, *n* (%)	36 (21.7)	21 (29.6)	15 (15.8)	0.033
Hypertension, *n* (%)	8 (4.8)	5 (7.0)	3 (3.2)	0.43
Heart failure, *n* (%)	4 (2.4)	0 (0)	4 (4.2)	0.14
Coronary artery disease, *n* (%)	7 (4.2)	5 (7.0)	2 (2.1)	0.24
Glucose (mmol/L)	7.5 ± 3.1	9.7 ± 3.1	5.9 ± 1.7	<0.001
Troponin (ng/ml)	7.1 (5.6, 9.6)	8.0 (5.9, 11.6)	6.2 (5.1, 8.1)	0.170
NT-proBNP (ng/L)	61.0 (25.5, 126.5)	41.0 (18.0, 92.5)	72.5 (40.0, 250.0)	0.012
TC (mmol/L)	4.8 ± 1.0	4.7 ± 1.0	4.9 ± 1.0	0.202
LDL-C (mmol/L)	2.7 ± 0.8	2.7 ± 0.8	2.7 ± 0.7	0.793

BMI, body mass index; TC, total cholesterol; LDL-C, low-density lipoprotein cholesterol.

Continuous data were presented as mean ± SD or median and interquartile range, and categorical data as a percentage of the sample.

### 3.2 Heart rate responses by T2DM

All exercise test results are shown in Table 2. At rest, patients with T2DM had higher systolic blood pressure, heart rate, and metabolic equivalents (METs) compared to those without T2DM, while their diastolic pressure was lower. However, these differences were not statistically significant. During the exercise load, patients with T2DM had a significantly lower peak heart rate than those without T2DM (*P* < 0.001). The peak systolic blood pressure and peak METs of patients with T2DM were lower than those of individuals without T2DM, while their peak diastolic pressure was higher, but these indicators were not statistically significant. The heart rate recovery for patients with T2DM was 14.65 ± 11.89 s, compared to 20.26 ± 8.58 s for those without T2DM (*P* < 0.001). In terms of chronotropic competence, the chronotropic index 2 for patients with T2DM was 7.88 ± 3.19, significantly lower than the 10.91 ± 2.75 for those without T2DM (*P* < 0.001). The chronotropic index 1 for patients with T2DM was also significantly lower than that for individuals without T2DM (*P* < 0.001). There were no significant differences between the two groups in VO2/Kg, RER, VC, or VEmax.

**Table 2:** Exercise test variables of the patients with and without T2DM.

Variable	T2DM	No-T2DM	*P*-value
Reasting systolic BP (mmHg)	117.50 ± 15.44	117.39 ± 14.00	0.96
Resting diastolic BP (mmHg)	71.63 ± 10.13	71.68 ± 11.75	0.98
Peak systolic BP (mmHg)	155.19 ± 26.91	162.96 ± 25.77	0.07
Peak diastolic BP (mmHg)	77.58 ± 13.01	75.62 ± 11.32	0.31
Reasting METs	1.45 ± 0.26	1.40 ± 0.27	0.26
Peak METs	5.99 ± 1.89	6.29 ± 1.33	0.22
Reasting HR (beats/min)	82.49 ± 12.89	80.43 ± 10.54	0.27
Peak HR (beats/min)	118.85 ± 23.66	133.26 ± 21.48	<0.001
Heart rate recovery (beats/min)	14.65 ± 11.89	20.26 ± 8.58	0.001
CI 1	0.46 ± 0.23	0.63 ± 0.21	<0.001
CI 2	7.88 ± 3.19	10.91 ± 2.75	<0.001
VO_2_/Kg (ml/min/kg)	20.95 ± 6.60	22.09 ± 4.80	0.22
VE/VCO_2_ slop	29.41 ± 2.86	28.07 ± 2.30	0.001
RER	1.12 ± 1.07	1.03 ± 0.09	0.41
VC (L)	3.01 ± 0.64	3.13 ± 0.86	0.33
FEV1/FVC	83.67 ± 12.66	3.13 ± 0.86	0.04
VEmax (L)	44.80 ± 18.41	46.39 ± 15.78	0.56

BP, blood pressure; METs, metabolic equivalents; CI, chronotropic index.

Continuous data were presented as mean ± SD.

Table 3 shows the impact of T2DM on chronotropic competence. Initial univariate logistic regression analyses were conducted to clarify the relationship between chronotropic competence and T2DM. This was followed by multivariate logistic regression analyses to exclude potential confounding factors affecting the relationship between chronotropic competence and T2DM. In the unadjusted Model 1, both chronotropic index 1 (OR: 0.023; 95% CI: 0.004–0.126; *P* < 0.001) and chronotropic index 2 (OR: 0.705; 95% CI: 0.620–0.801; *P* < 0.001) were negatively correlated with T2DM, with lower values of both indices associated with T2DM. Model 2, adjusted for age, gender, BMI, and smoking history, showed that the OR for chronotropic index 1 with T2DM was 0.043 (95% CI: 0.007–0.253; *P* < 0.001), while the OR for chronotropic index 2 with T2DM was 0.718 (95% CI: 0.621–0.831; *P* < 0.001). Model 3, building on Model 2, included adjustments for hypertension, heart failure, and coronary artery disease, and showed that the OR for chronotropic index 1 with T2DM was 0.062 (95% CI: 0.010–0.374; *P* < 0.001), while the OR for chronotropic index 2 with T2DM was 0.732 (95% CI: 0.630–0.852; *P* < 0.001). Model 4, based on Model 3, included additional adjustments for blood glucose, BNP, VO2/Kg, and VE/VCO2 slope, and the correlation remained significant. The chronotropic index 1 and chronotropic index 2 showed a consistent negative association with T2DM, remaining significant even after adjusting for various potential confounding factors. Subsequently, chronotropic index 2 was included in the model as a quartile variable. After adjusting for confounding factors, chronotropic index 2 still showed a negative correlation with T2DM (OR: 0.388; 95% CI: 0.173–0.869; *P* = 0.021), while the chronotropic index 1 itself was not associated with T2DM (OR: 0.414; 95% CI: 0.147–1.165; *P* = 0.095).

**Table 3:** Odds ratios and 95% CI of type 2 diabetes and chronotropic index.

Variable	Model 1	Model 2	Model 3	Model 4
CI 1	0.023 (0.004–0.126)*	0.043 (0.007–0.253)*	0.062 (0.010–0.374)*	0.001 (0.0001–0.521)*
CI 2	0.705 (0.620–0.801)*	0.718 (0.621–0.831)*	0.732 (0.630–0.852)*	0.665 (0.479–0.923)*
CI 1 group	0.522 (0.384–0.710)*	0.586 (0.424–0.808)*	0.634 (0.455–0.883)*	0.414 (0.147–1.165)
CI 2 group	0.374 (0.265–0.568)*	0.393 (0.265–0.582)*	0.415 (0.275–0.624)*	0.388 (0.173–0.869)*

CI, chronotropic index; CI group, the quartiles of the chronotropic index; Model 1: univariate logistic regression; Model 2: additionally adjusted for Model 1 variables plus age, sex, BMI, smoking history; Model3: additionally adjusted for Model 2 variables plus hypertension, heart failure, coronary artery disease; Model4: additionally adjusted for Model 3 variables plus Glucose, NT-proBNP, VO_2_/Kg, VE/VCO_2_ slop.

Data were presented as odds ratios and 95% confidence intervals.

* *P* < 0.05.

## 4 Discussion

Our study primarily found that patients with T2DM exhibited a lower heart rate response during exercise compared to individuals without T2DM. Even after adjusting for age, gender, BMI, smoking history, comorbidities, and other variables, this result remained unchanged. We also discovered that chronotropic index 2 is more closely related to T2DM than chronotropic index 1, with a lower chronotropic index 2 significantly associated with T2DM. These results remained consistent even after adjusting for other confounding factors.

We thoroughly analyzed heart rate response indicators in patients with T2DM, specifically focusing on their response during exercise. For the first time, we compared the change in heart rate per 1 MET increase during exercise between patients with T2DM and those without (chronotropic index 2). We confirmed that chronotropic index 2, as opposed to chronotropic index 1, is more closely related to T2DM and may more accurately assess the heart rate response of T2DM during exercise.

Previous studies have found that patients with diabetes may have impaired heart rate recovery. Seshadri et al. discovered that, in a healthy cohort without known coronary artery disease, diabetes was associated with abnormal heart rate recovery after exercise. This association persisted even after adjusting for several potential confounding factors (13). Yu et al. also found that delayed heart rate recovery after exercise is an independent risk factor for T2DM in men, even after adjusting for biochemical indicators such as glucose metabolism (14). These studies suggested that impaired heart rate recovery may be an early manifestation of diabetes and could predict the onset of diabetes. However, other studies have found that impaired heart rate recovery is not independently associated with the occurrence of diabetes. Jae et al. discovered that while slowed heart rate recovery is related to the development of T2DM, this relationship became insignificant after adjusting for diabetes risk factors and fasting blood glucose. They believed that this relationship could largely be explained by baseline fasting blood glucose in healthy males (15). Numerous studies have also found that patients with diabetes tend to have an increased resting heart rate. Park et al. found that for every 10 beats per minute increase in resting heart rate, the risk of diabetes increases by 1.39 times for men and 1.28 times for women (16). A meta-analysis shows a strong positive association between high resting heart rate and the risk of T2DM (17). A prospective cohort study conducted by Lee found that an increase of 10 beats per minute in resting heart rate is associated with a 19% increase in the risk of T2DM (18). Additionally, some studies have found the association between resting heart rate and diabetes to be unclear. An increase of 12 beats per minute in resting heart rate is associated with approximately a 10% higher risk of developing diabetes. However, this association becomes statistically insignificant after adjusting for BMI and postprandial blood glucose (19).

Heart rate recovery after exercise is primarily related to the activation of the parasympathetic nervous system, while the heart rate response during exercise is mainly associated with the activation of the sympathetic nervous system. Previous studies have shown that patients with diabetes exhibit significant sympathetic nervous responses during exercise, but the direction of this response varies across studies. Some studies have found that patients with T2DM have significantly enhanced sympathetic responses during isometric handgrip exercises (20). Additionally, animal experiments have shown that T2DM rats exhibit significantly stronger heart rate responses during muscle contraction and tendon stretch compared to healthy controls (21, 22). However, other studies have found weaker sympathetic activation responses in diabetic patients during exercise. Sydó et al. found in a cohort study of 21,396 patients without cardiovascular disease that diabetic patients had a lower chronotropic index, and this low heart rate response independently predicted long-term survival in diabetic patients (23). Our study similarly found a decrease in heart rate response during exercise in patients with T2DM, reflecting impaired sympathetic activation. It is noteworthy that our study is more comprehensive, especially focusing on T2DM patients, with a more detailed study design. The differences in these study results may be related to the choice of exercise mode in the studies—some focused on static exercises (e.g., handgrip tests), while others focused on dynamic exercises (e.g., aerobic exercise). Additionally, the duration of exercise may also be a factor influencing heart rate response. Whether the heart rate response is enhanced or diminished, it indicates abnormal cardiovascular responses during exercise in T2DM patients. This abnormal response may be an important marker of the increased cardiovascular risk in diabetic patients.

At any time, the heart rate reflects the function of the autonomic nervous system, which is the dynamic balance between the parasympathetic and sympathetic nerves. During exercise, sympathetic nerve tension increases and parasympathetic nerve tension decreases, leading to a gradual increase in heart rate (24, 25). Therefore, if the heart rate does not increase correspondingly with the intensity of exercise, it indicates a dysfunction of the autonomic nervous system (9).

The overt clinical symptoms of autonomic nervous system dysfunction in patients with T2DM often appear in the late stages, but subclinical dysfunction may already be present in the early stages. Hypotheses about the etiology of diabetic neuropathy include metabolic injury to nerve fibers, inadequate neurovascular function, autoimmune damage, and deficiency of neurotrophic growth factors. This pathogenic process involves multiple factors. Hyperglycemia leads to the accumulation of sorbitol and NAD by activating the polyol pathway. The activation of protein kinase C causes vasoconstriction and reduces nerve blood flow. Increased oxidative stress can cause endothelial damage and reduce the bioavailability of nitric oxide. Alternatively, excessive production of nitric oxide may lead to the formation of peroxynitrite and damage to the endothelium and neurons, a process known as nitric oxide stress. In the subgroup with neuropathy, immune mechanisms may also be involved. The reduction in nerve growth factor, deficiency of essential fatty acids, and the formation of advanced glycation end products also lead to decreased intraneural blood flow and nerve hypoxia, altering nerve function. The result of this multifactorial process may be the activation of ADP-ribosylation, leading to the depletion of ATP, which in turn causes cell necrosis and activates genes associated with neuronal injury (21, 22). Therefore, we can assess autonomic nervous dysfunction in patients with T2DM by evaluating their early chronotropic function status.

Our study also has several limitations. (1) This is a single-center study with a relatively small sample size, and we cannot avoid selection bias, which may limit the generalizability of the results. Additionally, our study subjects are Chinese, and larger studies are needed to extend the findings to other ethnic groups. Therefore, larger multi-center studies are necessary. (2) This is a cross-sectional study, which does not effectively establish the causal relationship between T2DM and chronotropic competence. Thus, large cohort studies are needed to further confirm this relationship. (3) Because this is retrospective data, some laboratory test results are missing. To minimize the impact of missing data, we used statistical methods for imputation, but this may still lead to some bias. Therefore, we should be more cautious when interpreting these related indicators. (4) Because of the retrospective design of this study, we did not consider the impact of the duration of T2DM, blood glucose control, and the use of antidiabetic medications on the results. These factors may influence heart rate responses in patients with T2DM.

## 5 Conclusion

Our study suggests that heart rate response is significantly reduced in patients with T2DM, and a low chronotropic index is independently associated with T2DM. This finding highlights the importance of monitoring heart rate dynamics as a potential strategy for managing and identifying diabetes. However, since our study may have limitations in establishing causality, further prospective studies and randomized controlled trials are recommended to investigate the causal relationships of these associations and determine their clinical significance in patients with T2DM.

## Data Availability

The original contributions presented in the study are included in the article/Supplementary Material, further inquiries can be directed to the corresponding author.

## References

[1] GBD 2021 Diabetes Collaborators. Global, regional, and national burden of diabetes from 1990 to 2021, with projections of prevalence to 2050: a systematic analysis for the global burden of disease study 2021. Lancet. (2023) 402:203–34. 10.1016/S0140-6736(23)01301-6 37356446 PMC10364581

[2] Vinik AI Maser RE Mitchell BD Freeman R. Diabetic autonomic neuropathy. Diabetes Care. (2003) 26:1553–79. 10.2337/diacare.26.5.1553 12716821

[3] Vinik AI Nevoret ML Casellini C Parson H. Diabetic neuropathy. Endocrinol Metab Clin North Am. (2013) 42:747–87. 10.1016/j.ecl.2013.06.001 24286949

[4] Cheng YJ Macera CA Church TS Blair SN. Heart rate reserve as a predictor of cardiovascular and all-cause mortality in men. Med Sci Sports Exerc. (2002) 34:1873–8. 10.1097/00005768-200212000-00003 12471290

[5] Feldman EL Callaghan BC Pop-Busui R Zochodne DW Wright DE Bennett DL Diabetic neuropathy. Nat Rev Dis Primers. (2019) 5:42. 10.1038/s41572-019-0097-9 31197183 PMC7096070

[6] Pfeifer MA Weinberg CR Cook DL Reenan A Halter JB Ensinck JW Autonomic neural dysfunction in recently diagnosed diabetic subjects. Diabetes Care. (1984) 7:447–53. 10.2337/diacare.7.5.447 6499637

[7] Balady GJ Arena R Sietsema K Myers J Coke L Fletcher GF Clinician’s guide to cardiopulmonary exercise testing in adults: a scientific statement from the American Heart Association. Circulation. (2010) 122:191–225. 10.1161/CIR.0b013e3181e52e69 20585013

[8] Lauer MS Okin PM Larson MG Evans JC Levy D. Impaired heart rate response to graded exercise. Prognostic implications of chronotropic incompetence in the Framingham heart study. Circulation. (1996) 93:1520–6. 10.1161/01.cir.93.8.1520 8608620

[9] Brubaker PH Kitzman DW. Chronotropic incompetence: causes, consequences, and management. Circulation. (2011) 123:1010–20. 10.1161/circulationaha.110.940577 21382903 PMC3065291

[10] Guazzi M Adams V Conraads V Halle M Mezzani A Vanhees L EACPR/AHA joint scientific statement. Clinical recommendations for cardiopulmonary exercise testing data assessment in specific patient populations. Eur Heart J. (2012) 33:2917–27. 10.1093/eurheartj/ehs221 22952138

[11] Gibbons RJ Balady GJ Bricker JT Chaitman BR Fletcher GF Froelicher VF ACC/AHA 2002 guideline update for exercise testing: summary article. A report of the American College of Cardiology/American Heart Association task force on practice guidelines (committee to update the 1997 exercise testing guidelines). J Am Coll Cardiol. (2002) 40:1531–40. 10.1016/s0735-1097(02)02164-2 12392846

[12] Gibbons RJ Balady GJ Beasley JW Bricker JT Duvernoy WF Froelicher VF ACC/AHA guidelines for exercise testing. A report of the American College of Cardiology/American Heart Association task force on practice guidelines (committee on exercise testing). J Am Coll Cardiol. (1997) 30:260–311. 10.1016/s0735-1097(97)00150-2 9207652

[13] Seshadri N Acharya N Lauer MS. Association of diabetes mellitus with abnormal heart rate recovery in patients without known coronary artery disease. Am J Cardiol. (2003) 91:108–11. 10.1016/s0002-9149(02)03014-x 12505588

[14] Yu TY Jee JH Bae JC Hong WJ Jin SM Kim JH Delayed heart rate recovery after exercise as a risk factor of incident type 2 diabetes mellitus after adjusting for glycometabolic parameters in men. Int J Cardiol. (2016) 221:17–22. 10.1016/j.ijcard.2016.06.149 27400291

[15] Jae SY Carnethon MR Heffernan KS Fernhall B Lee MK Park WH. Heart rate recovery after exercise and incidence of type 2 diabetes in men. Clin Auton Res. (2009) 19:189–92. 10.1007/s10286-009-0007-4 19370372

[16] Park DH Goo SY Hong SH Min JH Byeon JY Lee MK Prognostic value of resting heart rate in predicting undiagnosed diabetes in adults: Korean national health and nutrition examination survey 2008–2018. Nutr Metab Cardiovasc Dis. (2023) 33:141–50. 10.1016/j.numecd.2022.09.012 37074077

[17] Aune D Ó Hartaigh B Vatten LJ. Resting heart rate and the risk of type 2 diabetes: a systematic review and dose-response meta-analysis of cohort studies. Nutr Metab Cardiovasc Dis. (2015) 25:526–34. 10.1016/j.numecd.2015.02.008 25891962

[18] Lee DH de Rezende LFM Hu FB Jeon JY Giovannucci EL. Resting heart rate and risk of type 2 diabetes: a prospective cohort study and meta-analysis. Diabetes Metab Res Rev. (2019) 35(2):e3095. 10.1002/dmrr.3095 30378246 PMC6398339

[19] Carnethon MR Yan L Greenland P Garside DB Dyer AR Metzger B Resting heart rate in middle age and diabetes development in older age. Diabetes Care. (2008) 31:335–9. 10.2337/dc07-0874 17959868

[20] Vranish JR Holwerda SW Kaur J Fadel PJ. Augmented pressor and sympathoexcitatory responses to the onset of isometric handgrip in patients with type 2 diabetes. Am J Physiol Regul Integr Comp Physiol. (2020) 318 (2):R311–9. 10.1152/ajpregu.00109.2019 31823673

[21] Ishizawa R Kim HK Hotta N Iwamoto GA Mitchell JH Smith SA TRPV1 (transient receptor potential vanilloid 1) sensitization of skeletal muscle afferents in type 2 diabetic rats with hyperglycemia. Hypertension. (2021) 77 (4):1360–71. 10.1161/HYPERTENSIONAHA.120.15672 33641357 PMC8109832

[22] Kim HK Hotta N Ishizawa R Iwamoto GA Vongpatanasin W Mitchell JH Exaggerated pressor and sympathetic responses to stimulation of the mesencephalic locomotor region and exercise pressor reflex in type 2 diabetic rats. Am J Physiol Regul Integr Comp Physiol. (2019) 317 (2):R270–9. 10.1152/ajpregu.00061.2019 31091155

[23] Sydó N Sydó T Merkely B Carta KG Murphy JG Lopez-Jimenez F Impaired heart rate response to exercise in diabetes and its long-term significance. Mayo Clin Proc. (2016) 91:157–65. 10.1016/j.mayocp.2015.10.028 26769183

[24] Cernea S Raz I. Management of diabetic neuropathy. Metab Clin Exp. (2021) 123:154867. 10.1016/j.metabol.2021.154867 34411554

[25] Freeman JV Dewey FE Hadley DM Myers J Froelicher VF. Autonomic nervous system interaction with the cardiovascular system during exercise. Prog Cardiovasc Dis. (2006) 48:342–62. 10.1016/j.pcad.2005.11.003 16627049

