# Supplementary figures and images for: Reduced heart rate response to exercise in patients with type 2 diabetes
Source: Front Cardiovasc Med. 2025 Mar 31;12:1446675. doi: 10.3389/fcvm.2025.1446675 (PMC11994578; doi:10.3389/fcvm.2025.1446675)

### *Supplementary Material*

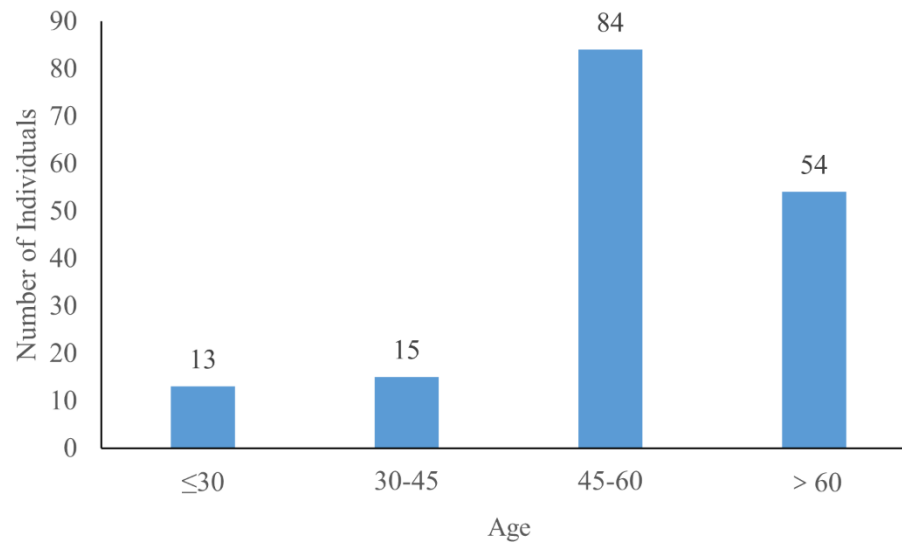

**Supplementary Figure 1.** Age distribution of participants.

Supplement: Supplementary file 1 [file Image1.pdf]
